# Reconstructing dynamical models from optogenetic data

**DOI:** 10.1186/1471-2202-16-S1-P143

**Published:** 2015-12-18

**Authors:** Sorinel A Oprisan, Patrick E Lynn, Tamas Tompa, Antonieta Lavin

**Affiliations:** 1 Department of Physics and Astronomy, College of Charleston, Charleston, SC 29424, USA; 2 Department of Computer Science, College of Charleston, Charleston, SC 29424, USA; 3 Department of Neuroscience, Medical University of South Charleston, Charleston, SC 29424, USA; 4 Faculty of Healthcare, Department of Preventive Medicine, University of Miskolc, Miskolc, Hungary

## 

Optogenetics allows optical control of neuronal activity by using genetically altered neural cells and optical tools. Briefly, optogenetics uses a photosensitive element that, upon absorption of light, produces some change in the activity of the cells. Although the technique evolved, it involves inserting a light-sensitive channel from green algae, called channelrhodopsin-2 (ChR2), into neurons [1]. By precise spatial and temporal delivery of light pulses we can identify the local interconnections among neurons and investigate their dynamical response under different conditions.

**Figure 1:**
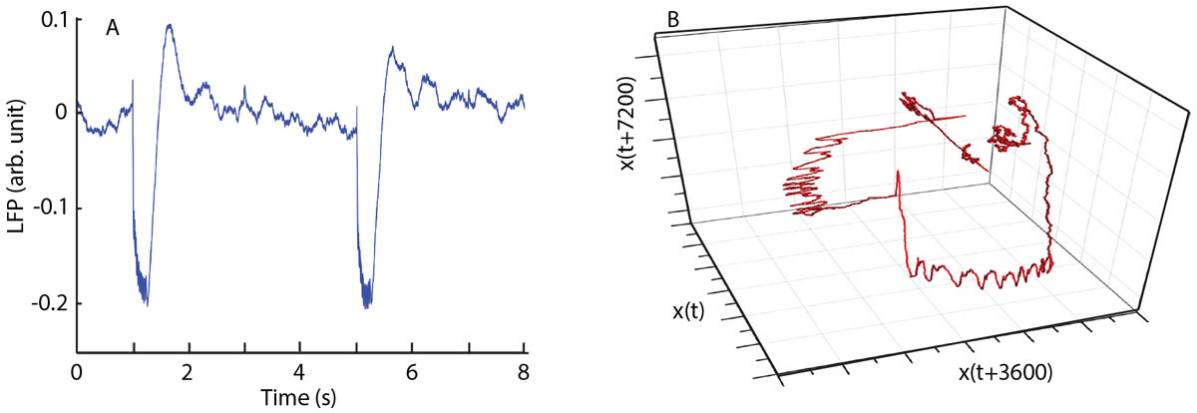
**A. Average local filed potential (LFP) recordings that show the response of mPFC to 250 ms light stimuli**. **B**. Phase space reconstruction of LFP using delayed embedding method in a three dimensional space and a delay time of 3600 data points (360 ms).

Data were recorded with a custom made optrode made of a recording pipette glued to optic fiber inserted in the medial prefrontal cortex (PFC) of male PV-Cre mice injected with virus suspension AV2/5.EF1a.DIO.hChR2(H134R)-EYFP.WPRE.hGH [2]. A 473 nm laser generated light stimulation and stable single unit recordings were monitored before filtering to record field potentials (0.1-100 Hz). Optical stimulation consisted of 10 ms pulses followed by 15 ms pause (40 Hz). A train of 10 pulses that lasted 250 ms was applied every 4 seconds and local filed potentials were recorded with a sampling rate of 10 KHz (Figure 1A). We used delay-embedding method to reconstruct the phase space attractor [3]. We found that the minimum dimension that unfolds the attractors is three and the delay time is about 3600 data points (Figure 1B). Based on the phase space reconstruction, we were able to extract a low-dimensional mathematical model that describes the dynamics of the system. Although every single neuron in the mPFC is described by a large number of independent variables such as ionic channel activation/inactivation variables, the local neural network activated by light pulses can be modeled with only three variables. We hypothesize these three global variables could be the activity of excitatory, inhibitory interneurons, and light-sensitive neurons.
